# Learning from the universal, proactive outreach of the Brazilian Community Health Worker model: impact of a Community Health and Wellbeing Worker initiative on vaccination, cancer screening and NHS health check uptake in a deprived community in the UK

**DOI:** 10.1186/s12913-023-10084-8

**Published:** 2023-10-12

**Authors:** Cornelia Junghans, Grazia Antonacci, Alison Williams, Matthew Harris

**Affiliations:** 1 Department of Primary Care and Public Health, School of Public Health, Imperial College London, 3Rd Floor Reynolds Building, St Dunstan’s Road, London, W6 8RP UK; 2 Centre for Health Economics and Policy Innovation (CHEPI), Business School, Imperial College London, London, UK

**Keywords:** Vaccination uptake, Screening uptake, Community Health Workers, Prevention, Hyperlocal, Reverse Innovation, Access to healthcare

## Abstract

**Background:**

Delays in preventative service uptake are increasing in the UK. Universal, comprehensive monthly outreach by Community Health and Wellbeing Workers (CHW), who are integrated at the GP practice and local authority, offer a promising alternative to general public health campaigns as it personalises health promotion and prevention of disease holistically at the household level. We sought to test the ability of this model, which is based on the Brazilian Family Health Strategy, to increase prevention uptake in the UK.

**Methods:**

Analysis of primary care patient records for 662 households that were allocated to five CHWWs from July 2021. Primary outcome was the Composite Referral Completion Indicator (CRCI), a measure of how many health promotion activities were received by members of a household relative to the ones that they were eligible for during the period July 2021-April 2022. The CRCI was compared between the intervention group (those who had received at least one visit) and the control group (allocated households that were yet to receive a visit). A secondary outcome was the number of GP visits in the intervention and control groups during the study period and compared to a year prior.

**Results:**

Intervention and control groups were largely comparable in terms of household occupancy and service eligibilities. A total of 2251 patients in 662 corresponding households were allocated to 5 CHWs and 160 households had received at least one visit during the intervention period. The remaining households were included in the control group. Overall service uptake was 40% higher in the intervention group compared to control group (CRCI: 0.21 ± 0.15 and 0.15 ± 0.19 respectively). Likelihood of immunisation uptake specifically was 47% higher and cancer screening and NHS Health Checks was 82% higher. The average number of GP consultations per household decreased by 7.4% in the intervention group over the first 10 months of the pilot compared to the 10 months preceding its start, compared with a 0.6% decrease in the control group.

**Conclusions:**

Despite the short study period these are promising findings in this deprived, traditionally hard to reach community and demonstrates potential for the Brazilian community health worker model to be impactful in the UK. Further analysis is needed to examine if this approach can reduce health inequalities and increase cost effectiveness of health promotion approaches.

**Supplementary Information:**

The online version contains supplementary material available at 10.1186/s12913-023-10084-8.

## Strengths and Limitations of the Study

o We examine the impact of a holistic proactive outreach model on uptake of vaccination, cancer screening and NHS health checks at household level in a deprived community. The Community Health and Wellbeing Workers (CHWWs) are based on the Brazilian Family Health Strategy which has scaled and seen remarkable improvements in Public Health over the years. This is the first evaluation in the UK of the CHWW role as delivered in Brazil.
o Our study used a new primary outcome measure, the uptake of prevention opportunities at household level (called the Composite Referral Completion Indicator—CRCI), offering possibilities as an outcome of interest in larger studies.
o We find encouraging positive outcomes in the completion of cancer screening, NHS health checks and vaccination uptake in those households that were visited by a CHWW compared to those households not yet visited. We also find a 7.4% reduction in unscheduled GP visits in these households compared to the year prior to the CHWWs becoming operational. Our study demonstrates a strong signal in this phase that the CHWW role will have wider population health level impact at scale.
o Although the CHWW programme has scaled into several other localities already, this study is only based on few interventions, across a short time period, covering only 662 households in a deprived ward in Westminster. The findings therefore might not be generalisable to other settings.

## Introduction

Primary prevention services such as immunisation and cancer screening have a substantial impact on morbidity and mortality [1], and lack of access or delays to these services will have important negative consequences over time for both individuals and the population, and potentially lead to inequalities. For example, over the last few years, the UK has experienced increases in vaccine preventable illness such as Measles, Mumps and Rubella [2, 3]. Many have discussed how delays in vaccination will lead to future outbreaks of pertussis [4], chickenpox [5, 6] and meningitis [7]. Low screening uptake for breast, cervical and bowel cancer, will lead to worsening mortality rates from late detection and treatment and certain groups are disproportionately impacted by this [8, 9]. Recent data show that just over a quarter of women invited for cervical screening don’t take it up [10] and this figure is even higher in women who are younger or come from deprived areas [11]. Even before the COVID-19 pandemic, immunisation, screening and health check uptake was at low levels [9, 12, 13].

Delays in uptake of these services has been attributed to lack of information and awareness or health literacy [13, 14] and solutions therefore typically include widespread public information campaigns [15]. But the evidence of effectiveness of these general strategies is unclear [16–18]. Non-targeted approaches can be harmful to some people [19] and widen disparities in uptake between different groups [17, 18]. Intensified efforts such as going to door-to door for COVID-19 vaccinator programmes in high prevalence areas with temporary recruited staff have shown demonstrable improvements in vaccination rates [20, 21], but these are specific to only some areas, only for specific vaccines, and are temporary and not scalable because the focus on just one vaccine type limits the economic justification for the role. Postal or SMS reminders have a place, but evidence of effectiveness is again equivocal [22]. Targeted cancer campaigns do increase uptake but there is evidence that people from ethnic minorities and sexual minorities are consistently underrepresented [23].

A recent report from the UK Wellcome Trust outlined effective strategies to increase vaccination uptake: (1) removing practical barriers by improving access, (2) rethinking communication about vaccines by avoiding ‘myth-busting’ and instead amplifying positive and accurate information and building resilience to false information, (3) presenting vaccination as a social norm and (4) inclusive research in different settings and populations [24]. Delayed uptake of any preventative service is more than simply lack of information or knowledge about those services. For example, families and households may not have health as their top priority, and concerns around housing, education or employment are all competing with preventative service uptake, particularly in households in deprived areas and particularly with recent rises in cost of living. Public information campaigns are important but will not help households tackle their more pressing priorities. Solutions therefore must be holistic and personalised, considering the household context. Improved uptake of immunization and screening services is the end result of a wide array of interventions, not necessarily overtly related to health, at the household level.

In a significant departure from the type of interventions typically delivered to respond to the issue of delayed uptake in preventative services, in 2021 Westminster City Council piloted a novel Community Health and Wellbeing Worker (CHWW) role that is universal, comprehensive and integrated into both primary care and the local authority. Inspired by the Brazilian Family Health Strategy [25–29], the key features are (1) local lay people, trained and paid, (2) practicing proportionately universal outreach by geographical area (approx. 150 households per CHW in a defined area) with monthly household visits irrespective of need or demand, (3) providing comprehensive support at the household level and (4) fully integrated into the primary care team and local authority. In Brazil, this approach has seen an impressive reduction of cardiovascular disease mortality of 34% and stroke mortality by 31% [30], 4.5% reduction in infant mortality [31] and reduction in horizontal inequity [32] because its scaling (by 2022 there were 250,000 CHWs across the whole country) provides comprehensive, household based regular and proactive support to over 70% of the population. Even lowest levels of coverage showed a statistically significant increase on women’s health, children’s health, diabetes and hypertension support [33]. In Brazil, the CHWs play a pivotal role in improving access to healthcare in the poorest regions, but also for the poorest people. There is good evidence to show that CHWs in Brazil reduce health inequities. Their dual roles, as healthcare workers but also community members, mean they can tailor messages, and be more relatable, than formally trained healthcare professionals. Spotting problems early on and supporting households to access care from any part of the system is an integral part of their effectiveness. CHWs are increasingly recognised to play an important role in the delivery of population-based primary care, particularly in the response to Covid-19 and vaccine hesitancy [34] and have been shown to have similar improvements in prevention opportunities in high-income countries [35]—although in western contexts they are often deployed in a more targeted, episodic and transactional community outreach role that is quite different from the universal, comprehensive and integrated approach seen in Brazil [36, 37].

**Fig. 1:**
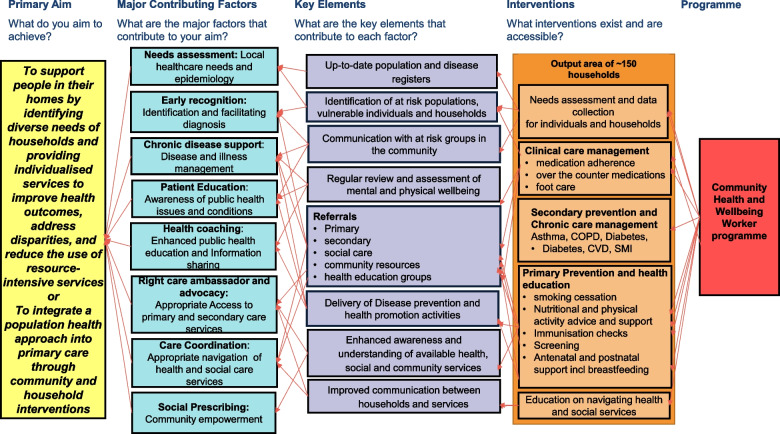
Programme Theory

Through regular proactive visits, and building trust with all households, the CHWs in Brazil are able to get to know the entire household, elicit social and health needs, discuss prevention opportunities in a personalised way, support chronic disease detection and management, be a first point of contact, signpost to services and resources in the community and connect with different professionals as needed (Fig. 1). In Brazil, their non-technical skills and attributes such as advocacy, civility and communication skills have been highlighted as major contributors to their success [38]. Based on the Brazilian experience, in this study we hypothesised that CHWWs would be able to identify anyone eligible for an immunization or screening service, explain and encourage uptake of that service in a timely manner as well as support the household in any other pressing issue that was interfering in the ability to prioritise their health. As a result of these wider, low-level but timely interventions, over time, for the households visited by CHWWs, there would be an improvement in uptake in primary prevention services that they are eligible for.

The Westminster pilot offered an opportunity to establish if CHWWs in the UK might be as effective at improving immunization and screening uptake as in the Brazilian context. The pilot in a deprived inner London borough began in 2021 with five part-time Community Health and Wellbeing Workers (CHWWs). Located in one of the most deprived social housing estates in the country, the CHWWs are residents in or near the estate, recruited by the local authority with an honorary contract with the local GP practice. The CHWWs are responsible for allocated buildings on the estate and, within these, households that are registered to the GP practice. The estate is in one of the worst performing wards for vaccine uptake nationally (78% MMR1, 85% DPT, 60% COVID1, 53% COVID2, 21% COVID Booster, 38% Reception age flu vaccine, 61% 65 + flu vaccine), has 61% social renting, a high proportion of BAME residents (49%) and recent migrants (28% households have no English speakers) [39]. The fact that it has taken time for CHWWs to reach all the households that they were originally allocated offers opportunity for a natural experiment and comparison in uptake of services between households that were visited and those that were not. This study ascertained whether there was an improved uptake of primary prevention services by individuals that were eligible for them, in households that were visited by the CHWWs compared to households that were not visited. Although the CHWWs deliver a whole array of health promotion activities (see Table 1), we focussed only on vaccinations, cancer screening and NHS health check uptake for this study.

**Table 1:** Examples of CHWW interventions in addition to standard care in the UK and outcomes included in this analysis vs outcomes not yet measured

Examples of CHWW interventions	Outcomes included in this analysis	Standard care	CHWW intervention in addition to standard care
Maternity and child health	Vaccination uptake only	Breastfeeding support in hospital or health visitor after birth	Information prior and hands-on breastfeeding support post labour, as and when needed
			Early encouragement to register for antenatal care, ongoing support throughout and after pregnancy, joined up care for other children, liaison with midwives, GP and health visitors, family navigators and others as needed
		Standard antenatal care: pt needs to register to receive 2 pregnancy ultrasound scans at 8–14 and 18–21 weeks, screening for certain conditions, 8 midwife and 1 GP visit (2 GP visits if first baby)	
			Signposting to antenatal and postnatal groups and classes
			Spotting domestic violence or safeguarding issues or children with special needs
		In some areas antenatal classes are offered (1 or 2 days)	
			Encouraging, explaining and facilitating developmental milestones reviews, vaccinations and screening opportunities such as chlamydia screening during pregnancy
		Newborn and mother wellness check after 6 weeks, blood spot, hearing test	
			BP checks in pregnant women
		Developmental milestones review by health visitor with ASQ-3 at 9–12 and 24 months	
		Vaccinations offered to mother during pregnancy and baby after birth and children according to vaccination schedule	
Cardiovascular disease and Diabetes	Only NHS health checks included	NHS health checks offered to anyone without known cardiovascular disease 40–70 yrs. of age every 5 years to check cholesterol, blood sugar, blood pressure, risk factors and signpost to appropriate services	Encouraging NHS health checks or other checks according to circumstances of resident, explaining blood pressure, taking blood pressure
			Promoting healthy lifestyles such as smoking cessation, healthy diet, physical activity in a personalised way
		If background of diabetes or cardiovascular disease relevant annual reviews and hospital checks e.g. diabetic retinopathy screening	
			Explaining medication and helping with compliance
Mental ill health	Not included	Severe mental ill health review	Recognising deterioration
		Services such as GP, single point of access crisis hotline, Talking therapy referral	Encouraging annual health checks
			Spotting antenatal /postnatal depression/ suicide prevention
			Supporting residents with learning disabilities
			Help with loneliness and isolation
			Service navigation
			Trained in Open Dialogue crisis intervention
Respiratory disease	Only vaccination uptake included	Asthma and COPD annual checks and care plans	Air pollution advice
			Help with inhalers/ explaining Asthma and COPD and care plans
		Flu and COVID-19, pneumococcal/ pertussis vaccinations for selected populations	
			Help with mould and damp in the house
			Check eligibility and encourage vaccine uptake
Cancer	Only vaccination and cancer screening uptake included	Cervical cancer screening 24–52 yrs. every 3 years, 53 to 64 every 5 years	Check eligibility and encourage cancer screening uptake
			Explain screening, facilitate booking
		Bowel cancer screening 60–74 yrs. every 2 years, in some areas 50 + , over 74 can request a test every 2 years	Explain red flags
			Encourage vaccinations e.g. HPV
			Lifestyle advice e.g. smoking cessation, promoting cancer IQ and self-checks
		Breast cancer screening for women aged 50–70 every 3 years, 71 + can request breast screening	
			Encourage booking with GP for PSA check if risk factors (e.g. black ethnicity)

## Materials and methods

CHWWs were assigned to around one third of all the households on the Churchill Gardens Estate and between July 2021 and January 2022 approximately 40% of the households allocated to the CHWWs had been visited at least once. Although the initiative is ongoing, for the purpose of this evaluation, we defined the intervention period as from July 2021 to January 2022 and allowed a lag of three months from the end of that intervention period for prevention opportunities to be taken up.

### Data

Data collected therefore covered the period July 2021 to April 2022. Pseudonymised records from the participating GP practice were analysed to compare the uptake of services that individuals were eligible for, in households that had received CHW visits compared to households that had been assigned a CHWW but that had not yet received a visit. Audits were run at the GP practice and de-identified for the purpose of exporting data from SystmOne, the electronic patient record system, and matching with those postcodes originally assigned a CHWW. Exported variables included whether households actively see a CHWW, and eligibility for all vaccinations, cancer screening and NHS health checks at the beginning of the intervention period. As a secondary measure, to assess whether CHWW visits were associated with increased or decreased demand for primary care services, GP appointments per household was also calculated using the year prior (2020) as a comparator. For details on how individuals were classified as eligible for each preventative service see Table 2.

**Table 2:** Individuals that were eligible (*n,* % total) and those who received services (*n,* % of those eligible for each service) in intervention and control groups

Service	Eligibility criteria in patients registered at the GP practice who received the intervention between 1^st^ July 2021 and 30^th^ April 2022^a^	N eligible individuals (% of total)	N eligible individuals (% of total)	*p*-value (Chi-squared test)	N individuals who received the service (% of total)	N individuals who received the service (% of total)	*p*-value (Chi-squared test)
		**Intervention**	**Control**		**Intervention**	**Control**	
**Immunisations**							
**COVID-19 (1**^**st**^** dose)**	> 12 yrs. as of 31^st^ Jan 2021. We assumed all individuals eligible for the vaccine given changes in eligibility criteria throughout the study period	608 (100%)	1643 (100%)	-	48 (8%)	94 (6%)	0.06
**COVID-19 (2**^**nd**^** dose)**		608 (100%)	1643 (100%)	-	97 (16%)	205 (12%)	0.03*
**COVID-19 (booster)**		608 (100%)	1643 (100%)	-	213 (35%)	376 (23%)	< 0.01*
**Shingles**	70–79 years of age (born between 1st Jul 1942 and 31st Jan 1952)	30 (5%)	68 (4%)	-	2 (7%)	4 (6%)	0.88
**DTaP/IPV/Hib/HepB**	2, 3 and 4 months (born between 1st Mar 2021 and 31st Jan 2022) only pts who received 3rd dose of vaccine included	1 (0%)	7 (0%)	0.41	0 (0%)	0 (0%)	-
**DTaP/IPV**	3 years and 4 months to 5 years (born between 1st Jul 2016 and 1st Oct 2018)	8 (1%)	23 (1%)	0.35	1 (13%)	4 (17%)	0.75
**Hib/MenC**	12 months to 3 years and 4 months (born between 1st Mar 2018 and 31st Jan 2021)	7 (1%)	28 (2%)	0.88	0 (0%)	3 (11%)	0.37
**HPV**	12–13 yrs. (born between 1st Jul 2008 and 31st Jan 2010)	12 (2%)	20 (1%)	0.35	1 (8%)	0 (0%)	0.19
**Influenza**	2–16 OR 65 + (born between 1st September 2005 and 31st March 2020 OR before 31st March 1957) or any age if in an at-risk group	173 (28%)	417 (25%)	0.18	49 (28%)	62 (15%)	< 0.01*
**MenB (3**^**rd**^** dose)**	12 months to 3 yrs. and 4 mns (born between 1st March 2018 and 31st January 2021)	7 (1%)	28 (2%)	0.14	0 (0%)	3 (11%)	0.37
**MMR (1**^**st**^** dose)**	1–5 yrs. (born between 30th Jun 2016 and 31st Jan 2021 who received the vaccines between 1st Jul 2021—30th Apr 2022	13 (2%)	46 (3%)	0.35	0 (0%)	3 (7%)	0.34
**MMR (2**^**nd**^** dose)**		13 (2%)	46 (3%)	0.16	0 (0%)	0 (0%)	-
**PCV (1**^**st**^** dose)**	3 months (born between 1st Apr 2021 and 31st Jan 2022)	1 (0%)	7 (0%)	0.38	1 (100%)	5 (71%)	0.54
**PCV (2**^**nd**^** dose)**	12 months (born between 30th June 2020 and 31st January 2021)	1 (0%)	5 (0%)	0.35	0 (0%)	3 (60%)	0.27
**PPV**	> 65 yrs. (born before 30th April 1957)	97 (16%)	247 (15%)	0.57	2 (2%)	1 (0%)	0.14
**Rotavirus**	2 and 3 months (born between 1st Apr 2021 and 31st Jan 2022) only pts who received 2^nd^ dose included	1 (0%)	7 (0%)	0.59	1 (100%)	5 (71%)	0.54
**Screenings and NHS Health Check**							
**Bowel cancer**	60–74 yrs., every 2 yrs., Last screening before July 2020. (born between 1st July 1947 and 31st January 1962)	39 (22%)	79 (16%)	0.11	12 (31%)	26 (33%)	0.81
**Breast cancer**	Women 50–71, every 3 years Last screening before July 2019 (born between 1st Jul 1950 and 31st Jan 1972	32 (18%)	65 (14%)	0.15	1 (3%)	0 (0%)	0.15
**Cervical cancer**	Women 25–49 years, every 3 years Last screening before July 2019. (born between 1st Jul 1972 and 31st Jan 1997)	81 (46%)	274 (57%)	0.01*	11 (14%)	25 (9%)	0.24
**Cervical cancer**	Women 50–64 years, every 5 years Last screening before July 2017. (born between 1st July 1957 and 31st January 1972)	43 (24%)	78 (16%)	0.02*	4 (9%)	8 (10%)	0.87
**NHS Health Check**	40–74 years, every 5 years^b^ Last NHS Health Check before July 2017. (born between 1st July 1947 and 31st January 1982)	51 (29%)	105 (22%)	0.07	18 (35%)	13 (12%)	< 0.01*

^*^Statistically significant result (p-value < *0.05). *

^a^Apart from influenza which must have been received between 1st September 2021—31st March 2022

^b^Exclusion criteria: statins prescription, hypercholesterolemia, atrial fibrillation, coronary heart disease, chronic kidney disease, diabetes, heart failure, hypertension, peripheral arterial disease, palliative care. Eligibility criteria for each service relative to the study period based on NHS standard criteria, and dates of service uptake. Based on criteria in: UK Health Security Agency. Complete routine immunisation schedule. London: UK Health Security Agency; 2022. Available from: https://assets.publishing.service.gov.uk/government/uploads/system/uploads/attachment_data/file/1055877/UKHSA-12155-routine-complete-immunisation-schedule_Feb2022.pdf [Accessed 21st June 2022]; NHS England. Screening and earlier diagnosis. Available from: https://www.england.nhs.uk/cancer/early-diagnosis/screening-and-earlier-diagnosis/ [Accessed 15th September 2022]. NHS. NHS Health Check. Available from: https://www.nhs.uk/conditions/nhs-health-check/ [Accessed 15th Sept

### Participants

Participants were eligible to be included in the analysis if they were currently residing in the estate, registered with the local GP practice who hosts the CHWWs in Westminster, and who had been assigned a CHWW at the beginning of the pilot (*n* = 2251 patients in 662 households) (Table 2). A household was defined as everyone living at the same address, including babies born to the household during the study period. Participants were split into intervention group (those that had received at least one visit by the CHWW) and control group (those assigned a CHWW but not yet received a visit by January 2022). All households had at least one individual eligible for one or more type of immunization, however not all households had individuals eligible for a screening intervention or NHS Health Check. Households where nobody was eligible for a screening intervention (*n* = 238) were excluded from the analysis of cancer screening, as individuals who are not eligible for screening should not receive it. Therefore, 120 households with 178 individuals were analysed in the intervention group for screening, and 304 households with 483 individuals were in the control group.

### Outcome variable

The primary outcome was the Composite Referral Completion Indicator (CRCI), a composite score of uptake of prevention opportunities at the household level, described in a previous article [40]. There are a few reasons why a combined primary outcome was chosen. Firstly, CHWWs could impact on any primary prevention opportunity, any vaccine type and any screening type so to focus on only one of these as a primary endpoint will risk missing the benefits found in uptake of other types of service. Secondly, including a raft of services in the outcome indicator increases the number of data points available within the relatively small pilot. Finally, CHWWs are deployed based on the number of households, not residents, they serve. A primary outcome measure that reflects outcomes seen for the entire household is a useful way to calculate the statistical power needed for a scaled CHWW research study, given CHWWs could impact on any number of preventative services [40]. We defined the CRCI as the proportion of vaccinations, cancer screening and NHS checks received by household members out of those that they were eligible to receive, based on standard NHS criteria and up to three months after the study period (i.e. 1^st^ July 2021 to 30^th^ April 2022) in order to take into account a reasonable time period for household members to obtain their immunizations or screening at the end of the specified intervention window (i.e. 1^st^ July 2021 to 31^st^ January 2022). The CHWWs were employed only from 1^st^ April 2021, so this intervention period represents a relatively early time point in their employment where they were just beginning to become established as CHWWs in the community, and where COVID19 lockdowns were still occurring through to November 2021, making home visits challenging.

We merged the list of patients with eligibility for a service as described above with the list of patients who had received a service within the study period. Details of codes are listed in the Additional file 1. We customised an unused READ code (Community Clinic Note) to ‘Community Clinic Note – has a Community Health and Wellbeing Worker’, to identify members of households actively visited. For each search, we exported the list of eligible patients and those who had received a service for further analysis after removing any patient identifiable information apart from the address. We then linked individuals to households by grouping all individuals with the same address and generating alphanumeric codes at the household level. Based on information from CHWW records, we were able to identify households that had been assigned a CHWW from the list of households registered at the GP practice (control group) and compare to those actively visited by a CHWW coded on S1 (intervention group). Households who had been assigned a CHWW but had not yet received a visit constituted the control group. We calculated the CRCI for each household and the mean and standard deviation of the overall CRCI in the intervention and control groups. We then calculated the CRCI for individual service categories (i.e., immunisations; screening and NHS Health Checks). Other variables comparing intervention and control group included household occupancy. We used Stata 13 for analysis.

A secondary outcome was the overall number of GP consultations received by patients in the intervention and controls group over the ten months before the start of the pilot (1^st^ September 2020 to 30^th^ June 2021), and during the first ten months of the pilot (1^st^ July 2021 to 30^th^ April 2022). For the analysis of GP consultations, we included patients who were actively registered at the GP practice at the time of the analysis. Consultations with a Locum, GP Partner, GP Registrar, GP Retainer, GP Sole Practitioner, GP Surgery, GP Trainee, GP/HV, GP CMO, GP Associate, GP Assistant, General Medical Practitioner, Clinical Practitioner Access Role were included unless they constituted scheduled reviews. For a list of SystmOne search codes used in the analysis refer to the Additional file 1.

### Statistical analysis

We calculated the CRCI for each household in the intervention and control groups. In the denominator, we included the total number of services that individuals in each household were eligible for during the intervention period. One individual could be eligible for multiple prevention opportunities. The numerator consisted of the total number of services received by individuals from the same household up to three months after the intervention period. We calculated the mean and standard deviation of the CRCI for each arm and compared them across the two groups. We show the CRCI in the intervention and control groups combined to measure uptake across all services at the household level as well as the CRCI for individual categories (immunisations; screening and NHS Health Checks).

As a result of missing data on eligibility, six households had a higher number of service records than those they appeared eligible for, which resulted in the CRCI being greater than 1. For these households, the CRCI was rounded to 1. Service eligibilities were used as proxies to compare the demographics of the intervention and control group, given that these are based on sex and age, as we were unable to extract patient identifiable information to directly compare sociodemographic characteristics of the two groups. The information on household occupancy was relevant because the number and demographics of individuals in each household, and therefore the number of services they are eligible for, have implications in terms of the effort required by CHWWs to have an impact on service uptake. The effort required to raise the CRCI in larger households might be less, because CHWWs would be able to communicate relevant information to more people in fewer visits, than for single-occupancy households. To assess whether any impact the CHWWs may have on primary prevention opportunity uptake could be due to improved recording rather than improved uptake, we also explored whether unscheduled GP appointments differed between the intervention and control groups with the hypothesis that CHWWs would reduce unscheduled appointments by resolving problems more quickly in the community. We therefore also carried out a difference-in-difference analysis on the number of GP consultations received by patients registered at the GP practice, as well as average number of GP consultations per patient and household, over the 10 months before the start of the CHWW initiative (1^st^ September 2020 to 30^th^ June 2021), and during the first 10 months of the pilot (1^st^ July 2021 to 30^th^ April 2022). In a sensitivity analysis, we excluded patients who had died or moved away.

### Ethics

Researchers responsible for data collection and analysis were provided with honorary contracts to work as S1 data analysts based at the GP practice and a data sharing agreement and Privacy Impact Assessment was obtained between the GP practice, Westminster City Council and Imperial College London. The data we used for this study was carefully de-identified and anonymised, to ensure no patient identifiable data was retained. As a service evaluation, ethics approval was not required. Public partners were not involved in the design or conduct of this study.

## Results

The populations of the intervention and control groups were largely comparable in terms of household occupancy and service eligibilities (Table 2) although there were slightly more large households in the intervention group compared to the control group (Table 3). All residents were eligible for vaccines given that everyone was included as eligible for the Covid-19 vaccine, however; not all households were eligible for a cancer screening or NHS health check, hence were excluded in that analysis.

**Table 3:** Number and proportion of households with varying occupancy in the intervention and control groups, for the “immunisations” and “screenings and NHS Health Check” household populations included in the analysis

	***Intervention group***	***Control group***	
***N of household members***	***N of households (% of total)***	***N of households (% of total)***	***p-value (Chi-squared test)***
***Immunisations***			
***Total***	*160 (100%)*	*502 (100%)*	*-*
*1*	*20 (13%)*	*144 (29%)*	< 0.01*
*2*	*35 (22%)*	*106 (21%)*	*0.84*
*3*	*26 (16%)*	*66 (13%)*	*0.32*
*4*	*21 (13%)*	*65 (13%)*	*0.95*
*5 or more*	*58 (36%)*	*121 (24%)*	< 0.01*
***Screenings and NHS Health Check***			
***Total***	*120 (100%)*	*304 (100%)*	*-*
*1*	*16 (13%)*	*40 (13%)*	*0.96*
*2*	*18 (15%)*	*55 (18%)*	*0.45*
*3*	*18 (15%)*	*46 (15%)*	*0.97*
*4*	*16 (13%)*	*56 (18%)*	*0.21*
*5 or more*	*52 (43%)*	*107 (35%)*	*0.12*

^*^ Statistically significant result (p-value < *0.05)*

### Overall service uptake

Early on in the pilot, one CHWW withdrew from the role, but all households allocated were still included in the analysis by being redistributed to the remaining four CHWWs. In total, 2251 patients in 662 corresponding households were allocated to the remaining four CHWWs and 160 households had received a visit during the intervention period. The remaining households, i.e., those that had not yet received a visit by a CHWW by January 2022 despite being assigned one, were included in the control group. Although these were not statistically significant findings, when looking at overall service uptake (i.e. immunisations, screenings, and NHS Health Checks combined), this was 40% higher among households that had received at least one visit by a CHWW (i.e. intervention group) compared to households that had been assigned a CHWW but had not yet received a visit (i.e. control group) (CRCI: 0.21 ± 0.15 and 0.15 ± 0.19 respectively) (Table 4). Immunisation uptake was 47% higher in the intervention group compared to the control group (CRCI: 0.22 ± 0.16 and 0.15 ± 0.18 respectively) (Table 4). The uptake of screenings and the NHS Health Check was 82% higher in the intervention group compared to the control group (CRCI: 0.20 ± 0.32 and 0.11 ± 0.26 respectively) (Table 4). The increase in immunization uptake was driven by a statistically significant 33% higher uptake among individuals in the intervention group compared with those in the control group of the second dose of the COVID-19 vaccine (16% and 12% respectively, *p*-value = 0.03), 52% higher uptake of the COVID-19 booster (35% and 23% respectively, *p*-value < 0.01), and 87% higher uptake of the influenza vaccine (28% and 15% respectively, *p*-value < 0.01) (Table 2). Immunisation uptake in the intervention group was equal to or higher than the control group for 8 of the 13 remaining immunisation categories (Table 2). The increase in screening and NHS Health Check uptake was driven by a 192% higher uptake of the NHS Health Check in the intervention group compared to the control group (35% and 12% respectively, *p*-value < 0.01) (Table 2). Screening uptake was higher in the intervention group compared to the control group for 2 of the 4 remaining screening categories, although not statistically significant (Table 2).

**Table 4:** Number of households in the intervention and control groups for different service categories included in the analysis. CRCI for intervention and control groups for overall service uptake (i.e., immunisations, screenings, and NHS Health Checks combined), as well as for immunisations, and screenings and NHS Health Check separately

	**Intervention group**		**Control group**	
Service category	**Households**	**CRCI (mean ± standard deviation)**	**Households**	**CRCI (mean ± standard deviation)**
Immunisations	160	0.22 (± 0.16)	502	0.15 (± 0.18)
Screenings and NHS Health Checks	120	0.20 (± 0.32)	304	0.11 (± 0.26)
*Overall*	***160***	***0.21 (*± *0.15)***	***502***	***0.15 (*± *0.19)***

### GP consultations

Before the intervention (Sep 2020 to Jun 2021), there were 144 households with 295 individuals who received the CHWW intervention 10 months later, and 262 households with 438 individuals who did not have at least one visit in the following 10 months. In total, 152 households with 301 individuals were in the intervention group from Jul 2021 until Apr 2022 and 271 households with 475 people in the control group. The overall number of GP consultations decreased by 2.2% in the intervention group over the first 10 months of the pilot compared to the 10 months preceding its start, whereas it increased by 2.9% in the control group over that same time period (Table 5). The average number of GP consultations per household decreased by 7.4% in the intervention group over the first 10 months of the pilot compared to the 10 months preceding its start, however it decreased by only 0.6% in the control group. (Table 5).

**Table 5:** Number of GP consultations, average GP consultations per household in the intervention and control group, before and during the CHW pilot

	**GP consultations**		**Average GP consultations per household**	
Period	**Intervention group**	**Control group**	**Intervention group**	**Control group**
Before intervention (Sep 20 – Jun 21)	**1441**	**1713**	10.01	6.54
During intervention (Jul 2021 – Apr 22)	**1409**	**1762**	9.27	6.50
% change	**-2.22%**	** + 2.86%**	**-7.39%**	**-0.61%**

In a sensitivity analysis we excluded patients who had been deducted from the GP practice register for various reasons (e.g. moved to a different address, passed away). The purpose was to explore the consistency of our findings, given that in the main analysis we were unable to determine the date in which patients no longer became actively registered at the GP practice, which possibly led to the overestimation of service eligibilities in the main analysis. Overall service uptake (i.e., immunisations, screenings, and NHS Health Check combined) was still 12% higher in the intervention group compared to the control group (CRCI: 0.29 ± 0.25 and 0.26 ± 0.27 respectively). Immunisation uptake was 16% higher in the intervention group compared to the control group (CRCI: 0.37 ± 0.27 and 0.32 ± 0.29 respectively); Screening and NHS Health Check uptake was 26% higher in the intervention group compared to the control group (CRCI: 0.24 ± 0.34 and 0.19 ± 0.34 respectively. This was driven by a 105% higher uptake of the NHS Health Check in the intervention group compared to the control group (41% and 20% respectively, *p*-value = 0.02).

## Discussion

### Findings in context

Our analysis has shown that a proactive outreach model in a community traditionally badged as ‘hard to reach’ led to a sizeable increase in the likelihood of uptake of delayed or missed vaccination and screening opportunities in those households visited at least once compared to households that had been allocated a CHWW but not visited during the intervention period. For several immunisation and screening types, and for NHS Health Checks, this was a statistically significant increase in likelihood of uptake which, despite the small study size and the relatively early stage in the deployment of the CHWWs during COVID lockdown, is particularly noteworthy. Despite the limited number of CHWWs working with a relatively small number of households over only six months of effective operations, some of which was impacted by the final COVD19 lockdown in the UK, this analysis suggests that they have been able to identify missed prevention opportunities and successfully refer and signpost them to have this done. Improved uptake of services such as immunisations, screening and NHS Health Checks will have an obvious, important downstream impact on health and care, and will address the entrenched inequities for this challenging context that has historically low uptake of services. As CHWWs increase their penetrance and reach into the community, and as they build stronger relationships with residents and allied professionals over time, it is reasonable to expect the CRCI to continue to increase compared to households that have not been allocated a CHWW. As of September 2022, CHWW engagement with their households was increasing with over 60% of their allocated households visited at least once.

A recent study, which extrapolated the findings in Brazil to the UK, modelled the likely impact on immunizations and screening uptake if every household in England was assigned a CHWW [28]. Assuming community health workers could engage with and successfully refer 20% of eligible unscreened or unimmunised individuals, it predicted an additional 753k cervical cancer screenings, 365k breast cancer screenings and 483k bowel cancer screenings, per annum. A total of 16k additional children annually could receive their MMR1 at 12 months and 25k their MMR2 at five years of age. This would have a salary cost of £2.2bn per annum [28]. However, if CHWWs were only funded in areas of high deprivation (e.g., in the 432 Primary Care Networks in the lowest 20% deprivation index) it would cost only £300m per annum which, based on previous findings [28], is the equivalent of the amount that unused prescriptions cost the NHS every year [40].

The CHWWs don’t only advise and support uptake of preventative services. They also offer chronic disease support, early detection of cognitive impairment, early antenatal support and postnatal support, monitoring developmental milestones in children particularly those not in a childcare setting, and also support with wider determinants of health, so the benefits of the role could reach far beyond the improved uptake of preventative services (see Table 1). Our analysis also showed that active visits by the CHWWs was associated with a decrease of average GP consultations by household in the intervention group when compared to the control group and the previous 10 months. Whilst we do not yet know whether the reduction of GP visits equates to better outcomes, if it does there is a compelling benefit also for overwhelmed primary care services, releasing capacity to attend to the needs of the sickest patients. Further analysis needs to explore whether CHWW input results in more appropriate consultations, better outcomes such as improved patient satisfaction or better health outcomes.

This study showed that in the early stage of implementation of the CHWW pilot, individuals eligible for services in households that were visited were 40% more likely to have received those services, compared to individuals eligible for the same services in households that were not yet visited.

### Strengths and limitations

The CHWW pilot is the first intervention of this kind in the UK, offering proactive monthly outreach by geography. Due to the relatively small number of individuals eligible for services and the limited number of households eligible for and seen by the service, numbers are small, and the evaluation was not powered to detect statistically significant differences between the groups. The CRCI is a composite measure, and its reliability has not been tested in larger populations. Multi-level regression modelling is needed to consider the effect of clustering at the household and GP practice level. A further issue may be that we do not know whether patients from the control group are still living at the same address, given CHWWs have not made contact them. Consequently, findings of CHWW impact may be a result of presence of “ghost patients” in the control group, rather than improvements of service uptake. However, this possible bias seems less likely given the analysis of GP consultations is showing significantly higher rates of GP activity in the control group. In addition, patients cannot be registered with more than one GP practice, so to have their screening done elsewhere they would have to do this privately or abroad, which is also less likely given the socio-economic profile of the population. The observed differences in uptake in primary prevention services could be due to improved recording at the GP practice for those households that have been visited by a CHWW. However, CHWWs do not directly enter data on screening and immunisation on the records - they merely encourage residents to take up services via the established routes.

Households in the control and intervention group are not allocated randomly, so it is not possible to assert that these observations are causally related to the CHWWs. Households that have received the CHWW visit may be systematically different to those that have not. Willingness to engage or respond to the CHWWs may in itself be associated with a greater willingness to engage with services. Alternatively, people who did not respond may be working longer hours and have issues accessing GP services including prevention opportunities. Sub-analysis shows minimal differences between the groups in terms of age and sex measured by proxy variables of service eligibility, and households do not differ significantly in terms of occupancy. However, it is possible that households who accepted a CHWW visit may be keener to take care of their health compared to those who did not engage. However, the residents who had not received a visit recorded more unscheduled GP contacts, hence it is unlikely that they are less concerned about their health, receive their prevention opportunities elsewhere or had difficulty accessing services. There may however be difficulty in access in terms of physical examination required for screening and GP engagement via a phone call, which we have not been able to assess.

Read codes on the clinical system are unreliably coded for different immunisation categories and this was particularly apparent with childhood immunisations, which looked spuriously low when compared to overall immunisation uptake figures. This may be explained by the fact that the CRCI only measures what prevention opportunities had been taken up within the study period. For example, someone who might be eligible for cervical screening within a 5-year period but had already had it 3 years ago would not be eligible for cervical screening for another 2 years and is therefore not included in the denominator. The CRCI measures only interventions that someone was eligible for, had not yet completed the intervention and subsequently received it in the study period, as we were interested in whether the CHWWs are able to motivate residents to take these up. Even if there were inconsistencies related to coding, this issue is likely to be equal in both the intervention and control groups. When looking at household occupancy, we assumed all individuals in each household were registered at the local GP practice that hosts the CHWWs, which may have led to the exclusion of individuals from the same household that were registered at different GP practices, and consequently an underestimation of the total number of service eligibilities in each household reported in this study.

For a small pilot evaluation, we were not able to assess the possibility of contamination between the control and intervention groups i.e., where the impact of the CHWW visits is felt also in unvisited households, through for example conversations between neighbours. However, any contamination is likely to bias the results towards the null hypothesis.

### Policy Implications

The impressive initial impact of CHWWs on service utilisation in households that they have visited provides a strong argument for continued investment in the role, including expanding its duration and scale. CHWWs build a longitudinal relationship with households, that is centred on trust and good communication. The ‘design code’ of the CHWW initiative that is important to preserve is fourfold: (i) hyperlocal, by paid and trained lay members of the community for their community, (ii) proportionately universal, i.e. not based on the ability or motivation to access services but actual observed need, (iii) comprehensive at the household level, including all ages and concerns and (iv) integrated in local authority, NHS and voluntary sector for maximum effectiveness. Applying proportionate universality is more likely to be effective in reducing health inequalities and the gap in life expectancy than a targeted approach by varying the scale and intensity of the universal action proportionate to the level of disadvantage [41].

### Research Implications

The real-world effect size demonstrated in this pilot will permit design of a suitably powered cluster-randomised controlled study. An intention-to-treat analysis would be appropriate comparing improvements in service uptake in the intervention group (which would include all households that have been assigned a CHWW, irrespective of whether they have received at least one visit or not), and the control group (that would include households that reside in the estate but have not been assigned a CHWW). A stepped-wedge design would assess the impact on the uptake of immunisations, screenings, and NHS Health Checks, as more initiatives are rolled out in different areas. Watt et al. [42] calculated that, based on power of 90% and 5% significance level, assuming 100 households per CHWW, and no similarity between households in terms of disease risk factors and compliance in uptake of interventions, a CRCI effect size of 30% could be demonstrated with 340 households (ICC = 0.01), 510 households (ICC = 0.02) and 1010 households (ICC = 0.05). For high levels of similarity in risk factors and compliance, the same effect size could be demonstrated with 500 households (ICC = 0.01), 750 households ((ICC = 0.02) and 1490 households (ICC = 0.05). Although this study was not powered to detect this, the CHWW role has since expanded into other localities across the UK, presenting opportunities for a suitably powered and controlled study to ascertain the impact of CHWWs at scale on uptake of preventative services. Initiatives such as the CHWW pilot are urgently needed if there is to be any impact on downstream population health outcomes such as vaccine-preventable illnesses, cancer mortality and cardiovascular disease. Furthermore, previous studies have shown that adoption of CHW models improve access to and equity in health care in high-income countries [43]. The increase in uptake of vaccinations and screening opportunities in this ‘hard to reach’ population with low levels up prevention uptake in our study may well represent a consequence of increased access and improved equity. Future studies need to confirm their direct impact on access and equity.

### Supplementary Information

**Additional file 1. **SystmOne search codes and additional search options for different service outcomes used to extract patient data for this analysis.

## Data Availability

The datasets used and/or analysed during the current study available from the corresponding author on reasonable request.
