# Supplementary material for: Learning from the universal, proactive outreach of the Brazilian Community Health Worker model: impact of a Community Health and Wellbeing Worker initiative on vaccination, cancer screening and NHS health check uptake in a deprived community in the UK
Source: BMC Health Serv Res. 2023 Oct 12;23:1092. doi: 10.1186/s12913-023-10084-8 (PMC10568890; doi:10.1186/s12913-023-10084-8)
Supplement: Supplementary file 1 — Additional file 1. SystmOne search codes and additional search options for different service outcomes used to extract patient data for this analysis. [file 12913_2023_10084_MOESM1_ESM.docx]

**Appendix**

SystmOne search codes and additional search options for different service outcomes used to extract patient data for this analysis.

| **Outcome** | **SystmOne search codes** |
| --- | --- |
| **Immunisations^a, b^** | |
| **COVID-19 vaccine (1st dose)** | Vaccination contents: SARS-2 Coronavirus, Part 1 |
| **COVID-19 vaccine (2nd dose)** | Vaccination contents: SARS-2 Coronavirus, Part 2 |
| **COVID-19 vaccine (booster)** | Vaccination contents: SARS-2 Coronavirus, Part Booster |
| **Influenza** | Vaccination contents: INFLUENZA |
| **MMR (1^st^ dose)** | Vaccination contents: RUBELLA, MEASLES, MUMPS, Part 1 |
| **MMR (2^nd^ dose)** | Vaccination contents: RUBELLA, MEASLES, MUMPS, Part 2nd Scheduled Booster |
| **DTaP/IPV/Hib/HepB** | Vaccination contents: DTaP/IPV/HiB/HepB, Part 3 |
| **Rotavirus** | Vaccination contents: ROTAVIRUS, Part 2 |
| **PCV (1^st^ dose)** | Vaccination contents: PNEUMOCOCCAL, Part 1 |
| **PCV (2^nd^ dose)** | Vaccination contents: PNEUMOCOCCAL, Part 2 |
| **PPV** | Vaccination contents: PNEUMOCOCCAL |
| **HiB/MenC** | Vaccination contents: MENINGOCOCCAL C, HIB |
| **MenB (3^rd^ dose)** | Vaccination contents: MENINGOCOCCAL B |
| **DTaP/IPV** | Vaccinations:   - Boostrix-IPV 1st Scheduled Booster - Boostrix-IPV 2nd Scheduled Booster - Boostrix-IPV Booster - DTaP/IPV 1 - dTaP/IPV 1 - DTaP/IPV 1st Scheduled Booster - dTaP/IPV 1st Scheduled Booster - DTaP/IPV 2 - dTaP/IPV 2 - DTaP/IPV 2nd Scheduled Booster - dTaP/IPV 2nd Scheduled Booster - DTaP/IPV 3 - dTaP/IPV 3 - DTaP/IPV 4 - DTaP/IPV Booster - dTaP/IPV Booster |
| **HPV vaccine** | Vaccination contents: HUMAN PAPILLOMAVIRUS |
| **Shingles** | Vaccination contents: VARICELLA-ZOSTER |
| **Screenings and NHS Health Check^a^** | |
| **Cervical cancer screening** | Read Codes: SMEAR (Cervical smearcodes) QOF cluster |
| **Cervical cancer screening** | Read Codes: SMEAR (Cervical smearcodes) QOF cluster |
| **Breast cancer screening** | Read Codes:   - Mammogram (537..) - Mammography normal (5372.) - Mammography abnormal (5373.) - Soft tissue X-ray breast NOS (537Z.) - Breast neoplasm screening normal (68620) - Breast neoplasm screening abnormal (68621) - Breast neoplasm screening NOS (6862Z) - Breast screening abnormal - told patient (9OH9.) - Breast screening administr.NOS (9OHZ.) - [D]Mammogram abnormal (R1380) - Mammogram - screening (X70ds) - Mammogram coned (X70dv) - Mammogram magnification (X70dw) - Xeromammogram (X70dx) - Breast neoplasm screening status (X74Wl) - Breast neoplasm screening (XE1TY) - Breast screening status (XaBTj) - Breast cancer detected by national screening programme (XaJU6) - Breast lump detected by mammogram (XaKit) - [V]Screening for malignant neoplasm of breast (ZV761) |
| **Bowel cancer screening** | Read Codes:   - Bowel cancer detected by national screening programme (XaN5C) - Bowel cancer screening programme: faecal occult blood result (XaPVj) - BCSP faecal occult blood test normal (XaPkd) - BCSP faecal occult blood test abnormal (XaPke) - Bowel scope (flexi-sig) screen: normal - no further action (XaabO) - Bowel scope (flx-sig) scrn: minor polyps removd -no f/u reqd (XaabP) - Bowel scope (flexi-sig) screen: referred for colonoscopy (XaabQ) - Bowel scope (flexible sigmoidoscopy) screen: cancer detected (XaabR) - Bowel scope (flexi-sig) screen: suspected cancer detected (XaabS) - Bowel scope (flexi-sig) screen: other abnormality detected (XaabT) - Bowel scope (flexi-sig) screen: incidental findings (XabUF) |
| **NHS Health Check** | Read Codes:   - NHS Health Check completed (523221000000100) - NHS Health Check programme (519961000000106) |
| **GP consultations** | Consultation method:   - Face to Face   Staff type:   - P Locum - GP Partner - GP Registrar - GP Retainer - GP Sole Practitioner - GP Surgery - GP Trainee - GP/HV - GP CMO - GP Associate - GP Assistant - General Medical Practitioner - Clinical Practitioner Access Role |

*^a^ Additional search option - Registration type: Registered for GMS, Applied for GMS.*

*^b^ Additional search option - Include vaccinations done under GMS, done under ‘other’ GMS, not done under GMS.*
